# Vagus Nerve Stimulation in the Carotid Triangle: An Effective Method for Monitoring the Recurrent Laryngeal Nerve in Thyroid and Parathyroid Surgery

**DOI:** 10.3390/jcm13010102

**Published:** 2023-12-24

**Authors:** Alfredo Traba, Angela de Abreu, Clara Nevado, Hipólito Duran, Antonio Gil, María Pérez Seoane, Laura Lopez-Gonzalez, Miguel A. Ortega, Melchor Álvarez-Mon, Pedro Martin, Juan San Roman, Raul Díaz-Pedrero

**Affiliations:** 1 Neurology and Neurophysiology, Hospital Universitario HM Sanchinarro, C/de Oña 10, 28050 Madrid, Spain; angeladeabreu@hmhospitales.com; 2 Neurology and Neurophysiology, Hospital Fundación Alcorcón, C/Budapest 1, 28922 Madrid, Spain; claranevado@hmhospitales.com; 3 General and Digestive Surgery Department, Hospital Universitario HM Sanchinarro, C/Oña 10, 28050 Madrid, Spain; hduran@hospitales.com; 4 General and Digestive Surgery Department, Hospital Universitario HM Montepríncipe, Av. de Montepríncipe 25, 28660 Madrid, Spain; info@drantoniogil.es (A.G.); mariapseoane@hmhospitales.com (M.P.S.); pmartinmarfil@hmhospitales.com (P.M.); 5 Ramón y Cajal Institute of Sanitary Research (IRYCIS), 28034 Madrid, Spain; laura.lgonzalez@uah.es (L.L.-G.); miguelangel.ortega@uah.es (M.A.O.); raul.diazp@uah.es (R.D.-P.); 6 Department of Surgery, Medical and Social Sciences, Faculty of Medicine and Health Sciences, University of Alcalá, 28801 Alcala de Henares, Spain; melchor.alvarezdemon@uah.es; 7 Department of Medicine and Medical Specialities, Faculty of Medicine and Health Sciences, University of Alcalá, 28801 Alcalá de Henares, Spain; 8 Immune System Diseases-Rheumatology and Internal Medicine Service, University Hospital Prince of Asturias, Networking Research Center on for Liver and Digestive Diseases (CIBEREHD), 28806 Alcalá de Henares, Spain; 9 General and Digestive Surgery Department, Hospital Universitario HM Torrelodones, Av. Castillo Olivares s/n, 28250 Madrid, Spain; juansanromandiego@hmhospitales.com; 10 General and Digestive Surgery Department, Hospital Universitario HM Rivas Vaciamadrid, 28521 Madrid, Spain

**Keywords:** vagus nerve stimulation in carotid triangle, recurrent laryngeal nerve monitoring, thyroid and parathyroid surgery

## Abstract

Objective: Our objective is the description of the technique of vagus nerve stimulation in carotid triangle in order to monitor the recurrent laryngeal nerve (RLN) during thyroid and parathyroid surgery. Methods: We stimulated the vagus nerve in the carotid triangle during 150 thyroid or parathyroid surgeries using a monopolar electromyography electrode inserted under the mastoid process towards the jugular foramen as a cathode, and using another subdermal electrode in the mastoid as an anode. Another complementary method of vagus stimulation was achieved with a pair of subdermal electrodes, placing the cathode at the mandibular angle and the anode at the mastoid. Results: In all patients, compound muscle action potential (CMAP) was recorded in the vocal cords with both stimulation techniques, allowing semi-continuous monitoring to be carried out. Intraoperative lesions were detected in 16 of the cases; 9 of them were transient with CMAP recovery achieved when modifying surgical maneuvers. Conclusions: Vagus nerve stimulation in the carotid triangle is a reliable technique for monitoring the RLN in thyroid surgery. Vagus nerve stimulation in the carotid triangle is effective and safe for RLN monitoring, and it is a clear alternative to direct continuous stimulation of the nerve that by contrast requires its dissection in the carotid sheath.

## 1. Introduction

The most serious intraoperative complication in thyroid surgery is recurrent laryngeal nerve (RLN) injury leading to vocal cord (VC) paralysis. This lesion has the potential to initially go unnoticed, being later detected only in the postoperative period when the patient presents a phonatory, swallowing or even respiratory difficulty in cases of a bilateral lesion. This circumstance led to the implementation of different nerve-control techniques, having been for many years the gold standard for the identification of the RLN in the surgical field used for its anatomical preservation [1]. However, even so, it is not uncommon for a nerve injury to occur in the presence of anatomical nerve integrity, produced by various mechanisms (traction, compression, thermal). The development of different neurophysiological techniques of intraoperative monitoring (IOM) of the RLN has allowed for its location during surgery; its functional preservation, with the continuous control of said function; and, in the case of a possible injury, the identification of the mechanisms needed to improve the surgical technique and establish a possible prognosis based on the type of damage produced [2].

Due to the indicated limitations of direct stimulation of the vagus nerve and the limited information that mapping in the surgical fields provides about the functional status of the laryngeal recurrent nerve, we decided to look for an alternative accessible, non-invasive and effective vagal stimulation technique to evaluate real-time nerve function

A major difficulty in the continuous monitoring of the RLN lies in the stimulation of the vagus nerve, which allows us to obtain compound muscle action potentials (CMAP) recorded directly in the VCs. This stimulation is usually performed with an electrode placed directly on the nerve, which in turn requires dissection of the vagus nerve in the carotid sheath inside the neck. As this technique also necessitates a larger surgical incision, and possible new complications, many surgeons do not perform this approach. We, therefore, present a technique for electrical stimulation of the vagus nerve in the carotid triangle (ESVNCT), with recording of the CMAP in VC and with minimal interference in the surgical activity, thereby allowing for semi-continuous monitoring of the functional integrity of the RLN.

## 2. Patients and Methods

### 2.1. Patients

We carried out a retrospective analysis on a series of patients who had undergone thyroid or parathyroid gland operations with the General Surgery teams at the HM Hospitals of Madrid. In each case, ESVNCT was performed, regardless of the use of other intraoperative monitoring techniques. Patients with a previous RLN injury were excluded from this study, as well as those cases in which the IOM was interfered with by technical factors that prevented the correct interpretation of the CMAP, usually due to malfunction of the recording electrode. Neither the age of the patients, the type of thyroid, or parathyroid pathology, nor the existence of previous surgeries without recurrent nerve injury were considered as exclusion factors, since our objective is to demonstrate the usefulness of vagal stimulation in all cases. The study has been approved by the Ethics Committee of the HM Hospitals of Madrid. Patients were included from January 2018 to March 2020.

### 2.2. Methods

IOM was performed using 16-channel Xltek Protektor equipment in all patients, and a combination of different techniques including continuous electromyography (cEMG), RLN, or vagus nerve mapping in the surgical field, and ESVNCT were used. Train of Four monitoring was also performed in all cases to rule out the existence of a possible neuromuscular blockade.

**Figure 1:**
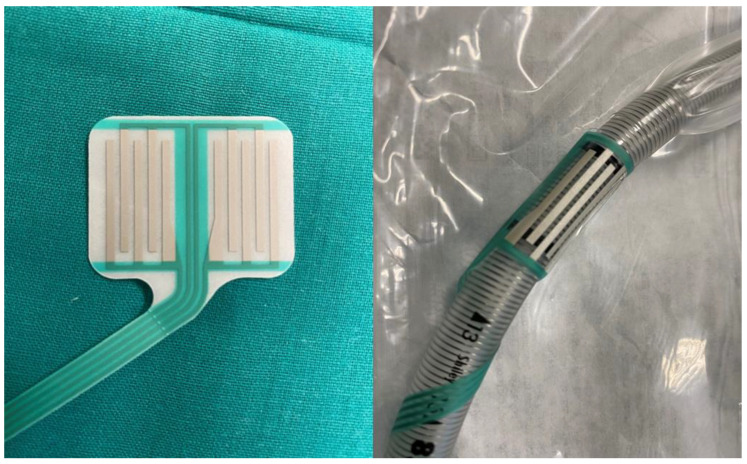
(**Left**) Circular surface electrode used to record EMG activity and CMAP on vocal cords. (**Right**) Circular surface electrode attached to the orotracheal tube.

The cEMG was performed using a circular surface electrode attached to the orotracheal tube and placed by the anesthesiologist at the level of the VC (Figure 1). Once placed in this position, the electrode allows us to record both spontaneous activity and activity caused by different stimulation methods. It is important to ensure that, after placing the neck in hyperextension, the recording electrodes are perfectly located at the VC level. For the recording of the cEMG, we used a sweep of 400 ms/div, with an amplitude of 100 µV/div and filters of 3–2000 Hz. With these recording parameters, we can easily identify different types of discharges and also differentiate them from artifacts induced by tracheal movement produced during thyroid dissection or through the use of different surgical instruments such as bipolar coagulation. This type of VC surface recording electrode has an important limitation though that we must always keep in mind. Although theoretically designed to record separate activity generated in the VC on each side, the circular distribution of recording points make it very difficult to differentiate them both in spontaneous cEMG and in CMAP after unilateral nerve stimulation, although in this case, higher amplitude responses have been described in the stimulation side [3]. In contrast, it does have the advantage of allowing CMAP to be obtained from the cricothyroid muscle after stimulation of the superior laryngeal nerve. Simultaneously, we performed a cEMG recording using subdermal electrodes placed bilaterally in the trapezius superioris (TS) muscle. The purpose of this recording is, among others, to confirm that the recorded activities originated in the VC and are not due to any possible electrical interference that would also alter the TS recordings.

Mapping in the surgical field of the RLN is used to identify the nerve and check its correct function with the use of a monopolar stimulation electrode placed directly by the surgeon on the various different structures that could potentially be confused with the nerve, thus checking if it is nerve tissue or not. By electrically stimulating the RLN, we can record CMAP in VC, confirming that the stimulated tissue corresponds to the nerve. During thyroid dissection, the surgeon primarily seeks visual identification of the RLN, which is essential for its preservation. Mapping can confirm that it is indeed the nerve, but sometimes there are vessels or tracts that, due to their appearance, could be confused with nerve fibers. In these cases, the mapping acquires fundamental importance for the correct identification of each structure. Mapping is essential in cases where there are anatomical variations of the RLN or in the presence of infiltrating tumors that completely distort the normal anatomy. To perform RLN mapping, the stimulator used by the surgeon is insulated, except at the tip, and acts as a cathode with the anode, and then act as a subdermal needle electrode placed on the clavicle or sternum. We use a constant current stimuli of 0.2 ms duration, with a frequency of 2–3 Hz and an initial intensity of 2 mA. If this intensity does not obtain CMAP in VC, we consider that the stimulated structure does not correspond to the RLN; if CMAP is obtained on the other hand, we progressively reduce the stimulation intensity until the motor threshold is determined and we confirm that it really is the RLN that is being directly stimulated. On occasions where it is difficult to locate the nerve, mapping can be completed using higher stimulation intensities, which has already been shown to not cause any harmful effects [4]. A 3–5 ms/div sweep, 0.2 mV/div gain and 20–3000 Hz filters are used to record CMAP in VC. In the surgical field, the vagus nerve can also be mapped, even if it is not exposed, since the stimulator can be placed in the proximity of the carotid artery under the sternocleidomastoid muscle, recording CMAP in VC with a greater latency than that obtained through activation of the RLN, and it allows us to confirm the integrity of the nerve throughout its course. Another way of confirming the integrity of the RLN is through mapping once the thyroidectomy is complete, stimulating the nerve at the most distal point (nerve entrance in the larynx) as well as at the most proximal in the surgical field, and then comparing the amplitude of the CMAP from both points. The direct stimulation of the RLN always produces CMAP in the recording of both VCs, so the differentiation between the VCs is given using the stimulation side but not the EMG recording.

**Figure 2:**
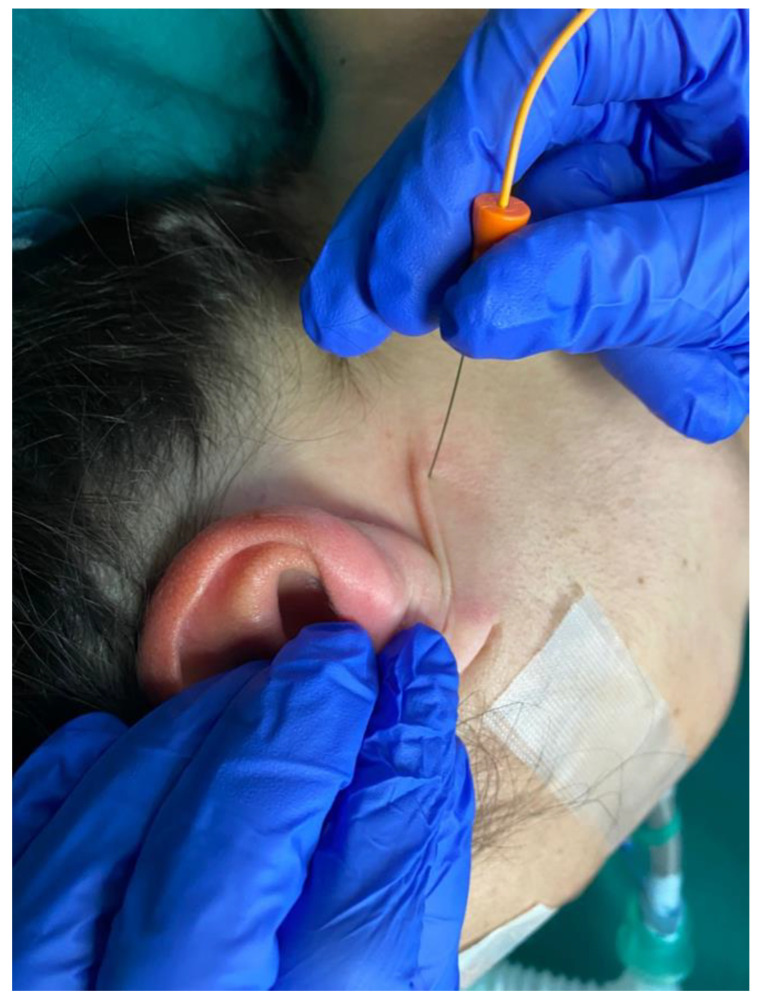
Stimulation of the vagus nerve in carotid triangle. A monopolar electrode, used as cathode, is inserted below the mastoids directed inwards and forwards towards the jugular foramen.

**Figure 3:**
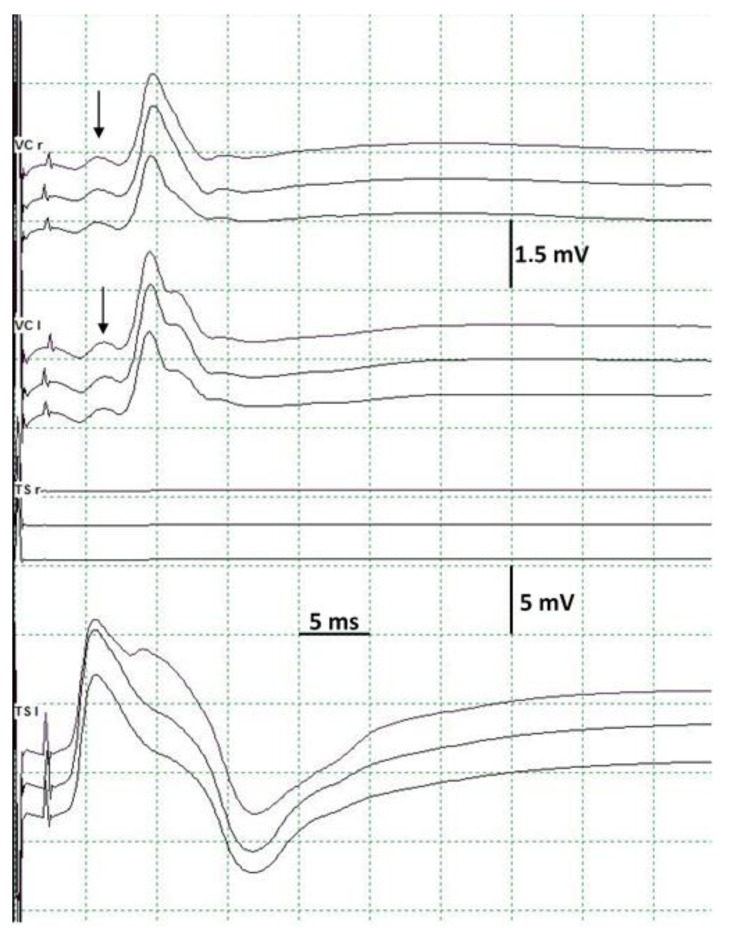
Normal CMAP recorded bilaterally in vocal cords after left unilateral stimulation. CMAP is recorded in left trapezius superioris muscle but not in the right, showing that there is no diffusion contralateral to the stimulus. Note, there is a small early potential (arrows) that corresponds to the CMAP of superior laryngeal nerve generated in cricothyroid muscle. VC: vocal cord. TS: trapezius superioris. r: right. l: left.

The main finding presented by our IOM is the ESVNCT, which is achievable without the need for dissection of the vagus nerve in the carotid sheath, thereby allowing for the thyroidectomy to be achieved through a smaller incision. After leaving the skull through the jugular foramen, together with the spinal and glossopharyngeal nerves, the vagus nerve follows a descending path through the carotid triangle in a close relationship with the carotid artery and the internal jugular vein. The vagus nerve stimulation is carried out in two different ways, which complement each other and also give us greater security in monitoring. One of the stimulation techniques consists of a monopolar EMG needle electrode introduced below the mastoids directed inwards and forwards towards the jugular foramen, and used as a cathode with another subdermal electrode located in the mastoids acting as an anode (Figure 2). In the other technique, we use subdermal needle electrodes located immediately caudal to the mandibular angle (cathode) and in the mastoid process (anode). With this arrangement, we stimulate the vagus nerve as it passes through the cervical carotid triangle. A constant current with single stimuli of 0.2–0.5 ms duration is applied in both cases, recording the CMAP with the orotracheal tube electrode, establishing baseline responses prior to the start of the intervention. This stimulation is performed semi-continuously throughout the entire surgery, repeating itself every few minutes to ensure normal function of the RLN, and, in general, always before and after performing any risky maneuver or at the specific request of the surgeon. The stimulation is very fast, barely interrupting the surgery by 1–2 sg. For the CMAP recording we use 5 ms/div sweep, with an initial gain of 0.2 mV/div that varies depending on the CMAP amplitude and 10–3000 Hz filters. Sometimes the stimulation artifact can include the initial part of the CMAP, making it difficult to determine its latency. In such cases, the low-frequency filters are modified until the artifact is attenuated and the perfect beginning identification for the CMAP is achieved. The intensity of stimulation used depends on the anatomy of the patient’s neck, but it is always necessary to ensure that the stimulus is supramaximal. The stimulation intensity is always lower with the monopolar EMG electrode if it is correctly located due to its greater proximity to the nerve than with percutaneous stimulation, being even more evident in patients with thicker necks. With these stimulation methods, we can also simultaneously activate the spinal nerve, as it has a course very close to that of the vagus nerve in its most proximal extra-cranial segments, recording the CMAP in the TS muscle. In this way, we ensure the efficacy of the stimulation through obtaining CMAP in muscle territories of different nerves. It is necessary to record the TS CMAP on both sides to control the possible current diffusion to contralateral nerves in case of using an intensity that is too high, as can occur in patients with very thin necks (Figure 3). With this stimulation, we also directly activate the chewing muscles due to their proximity to the cathode, so we must always take care to protect against tongue or lip injuries, or the biting of the orotracheal tube.

To perform this IOM, gas or total intravenous anesthesia (TIVA) anesthesia can be used indistinctly, depending on the anesthetist’s preference, since the stimulation we perform is always only on the peripheral nerve and is, therefore, not influenced by the drug used. To rule out the presence of a neuromuscular blockade, we perform a Train of Four in an upper extremity via the stimulation of the median nerve in the wrist and a recording of the CMAP in the abductor pollicis brevis muscle. For intubation, the anesthetist usually uses relaxants with a short half-life, whose effects disappear in a few minutes.

Statistical analysis of the results was performed to calculate the sensitivity, specificity, positive predictive value (PPV), and negative predictive value (NPV) of vagus nerve stimulation, comparing postoperative clinical courses with intraoperative CMAP changes.

## 3. Results

A total of 150 RLNs have been monitored in 95 patients, 55 of the cases being bilateral interventions, and the other 40 unilateral. The mean age of patients was 51.4 years with a range of 24–82 years. A total of 71.58% of the cases were female (68 patients) and 28.42% male (27 patients). In 86 cases, the patients were undergoing their first intervention, while the other 9 had been operated on previously. Unilateral interventions were right sided in 23 cases and left sided in 17 cases. Goiter was the most frequent etiology, with 33 cases (34.74%), followed by isolated nodules in 25 cases (26.32%), carcinoma in 20 cases (21.05%), parathyroid adenoma in 12 cases (12.63%), hyperthyroidism in 4 cases (4.21%) and isolated cervical lymphadenectomy in 1 case (1.05%). In seven patients, the thyroidectomy was associated with cervical lymphadenectomy, and in two others the goiter was intrathoracic.

**Figure 4:**
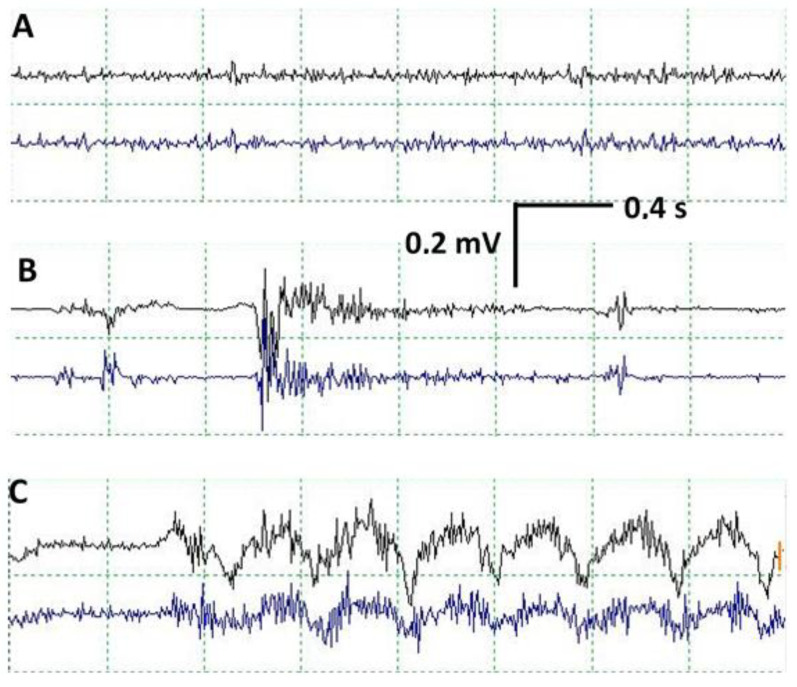
In each figure, the upper trace corresponds to the recording in the right vocal cord, and the lower trace corresponds to the left vocal cord. (**A**) Normal EMG recording in vocal cords. Continuous activity due to tonic contraction of vocal cords. (**B**) EMG discharge related to RLN manipulation. (**C**) Movement artifact due to displacement of the larynx during thyroid dissection, which can provoke changes in the morphology of the CMAP in VC due to movement of the orotracheal tube.

cEMG was performed for all patients, recording a continuous basal activity of low amplitude in relation to the muscle tone maintained by the VC. Superimposed EMG discharges of variable duration and intensity have been recorded in all patients in relation to RLN manipulation, mainly using traction. When this occurs, it should always be reported immediately to avoid possible nerve injury. Vagal stimulation should also be performed to check how conduction through the RLN is maintained, thereby ruling out any possible injury. We have not seen any cases of nerve injury associated with discharges in cEMG if it is not linked to abnormal changes in CMAP. Another type of activity that we recorded are movement artifacts due to the displacement of the larynx during thyroid dissection (Figure 4).

The surgeon did not perform intra-operative mapping of the RLN in 37 nerves, although he had the option to do so. Mapping through stimulating the most proximal and the most distal point of the nerve in the surgical field postoperatively was performed on 22 nerves, as was stimulation of the vagus nerve directly near the carotid artery to confirm the persistence of postoperative nerve conduction in 12.

ESVNCT was performed in all patients, and baseline CMAP was obtained with both types of vagal stimulation in all cases. Latency between 7 and 11 ms was noted on the right side, and 9.8–12 ms on the left, using mandibular angle stimulation; with stimulation close to the jugular foramen, the CMAP had a latency of around 0.5 ms longer due to more proximal nerve activation. The mean amplitude of the CMAP was 570.9 µV. Only one patient had low-amplitude responses that did not improve with repositioning of the orotracheal tube, but that did not prevent IOM from being performed. Likewise, the presence of a potential latency between 3 and 5 ms, anterior to the CMAP of the recurrent nerve was observed with the ESVNCT: simultaneously recording the potentials obtained with the orotracheal tube electrode and with needle electrodes placed in the cricothyroid muscle, it was verified that this potential corresponds to the CMAP of the superior laryngeal nerve, through stimulation of its external branch located in the carotid triangle (Figure 3). Of the 150 RLNs, vagal stimulation throughout the surgery was normal in 134 (89.33%) of those cases. Alteration in the CMAP, however, was noted in 16 of the procedures (10.67%), with unilateral alterations in 15 (93.75%), and bilateral in only 1. The CMAP alterations consisted of total loss of response in 4 nerves or partial (greater than 50% reduction [5]) in 12 nerves. Of the cases with total loss, three of them had no recovery of CMAP, presenting postoperative RLN paralysis confirmed using fiberoptic laryngoscope. In the remaining case, RLN paresis was confirmed and showed improvement after one month. Of the twelve nerves with partial loss of the CMAP, three had a severe reduction with minimal or no intraoperative recovery, one showed full recovery from RLN paralysis after nine months, and another displayed a transient dysphonia. The remaining patient presented postoperative respiratory difficulties requiring tracheostomy due to paralysis of the left VC and paresis of the right, though no significant changes in CMAP on this side were noted. After four months, the right VC was normal with stiffness in the left. The remaining nine cases with partial loss of the CMAP presented a significant (greater than 50%) or complete recovery intraoperatively, and none presented voice disorders. The incidence of RLN injury in our series is 5.33%. The alterations described in RLN CMAP were detected using both types of vagus nerve stimulation.

In three cases, the RLN was mapped in the surgical field with stimulation along its entire course. This mapping allowed for the location of the exact point where the CMAP was being lost, despite the anatomical integrity of the nerve. In all three cases, there was paralysis of the RLN with clear improvement after several months.

**Figure 5:**
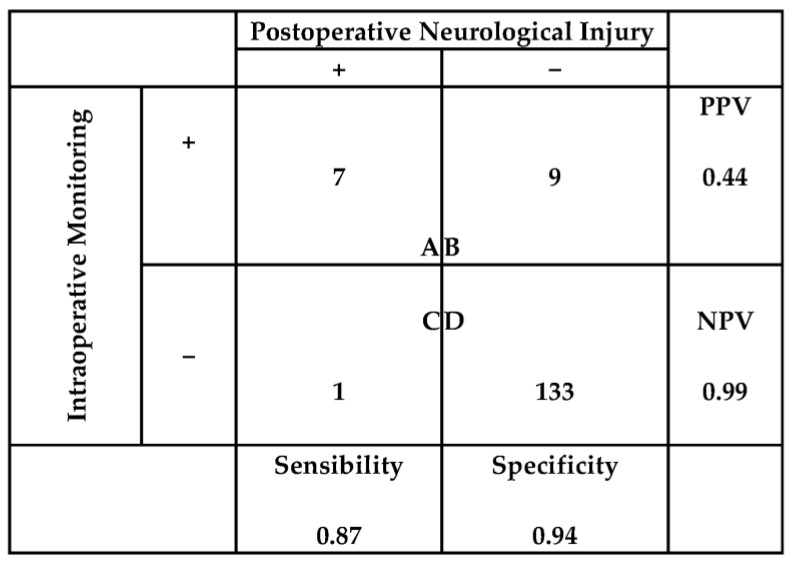
Statistical analysis of the results of vagal nerve stimulation in the carotid triangle to detect RLN lesions. PPV: positive predictive value. NPV: negative predictive value. (A) True positives. (B) False positives. (C) False negatives. (D) True negatives.

The statistical analysis of these results is reflected in Figure 5, where we can see that ESVNCT shows a sensitivity of 0.87 and a specificity of 0.94 to detect intraoperative RLN lesions, with a high NPV of 0.99, but very low PPV of 0.44.

**Figure 6:**
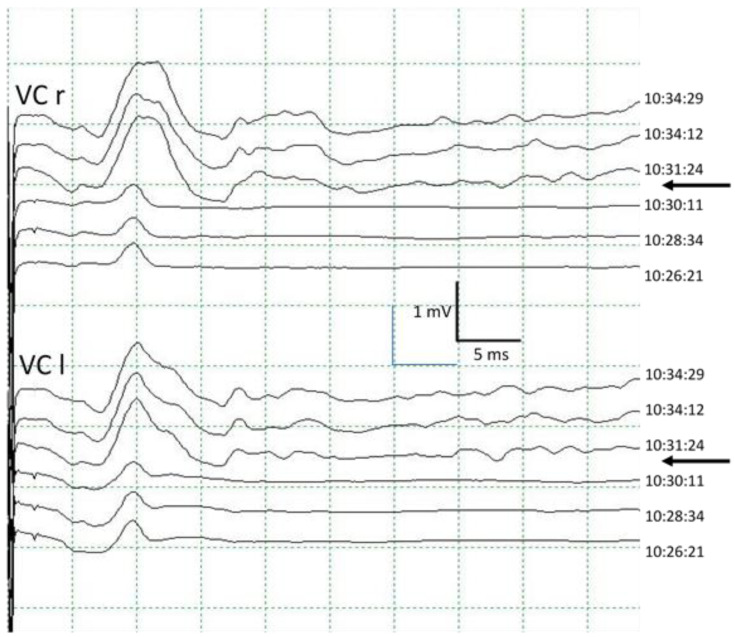
Immediate change in the morphology of the CMAP in the vocal cords when the orotracheal tube is repositioned (arrows), producing a significant increase in its amplitude. VC: vocal cord. r: right. l: left.

The morphology of the CMAP obtained in VC after ESVNCT has the same behavior with both stimulation techniques. It is variable, bi, or polyphasic. This variability is probably due to the characteristics of the recording electrode placed in the orotracheal tube. It presents several recording points located longitudinally allowing, theoretically, for the differentiation between the right and left side. In practice, however, this is not always the case, obtaining instead bilateral CMAP with unilateral stimulation (Figure 3). During the dissection of the thyroid gland, displacement of the larynx occurs very frequently, which we recorded in the cEMG as a movement artifact. This can also result in small movements of the tube from rotation or sliding and changes in the spatial relationship of the electrode recording areas with the VC, which results in changes in the CMAP both in morphology and amplitude. This should not be confused with signs of RLN injury [6] (Figure 6).

## 4. Discussion

**Figure 7:**
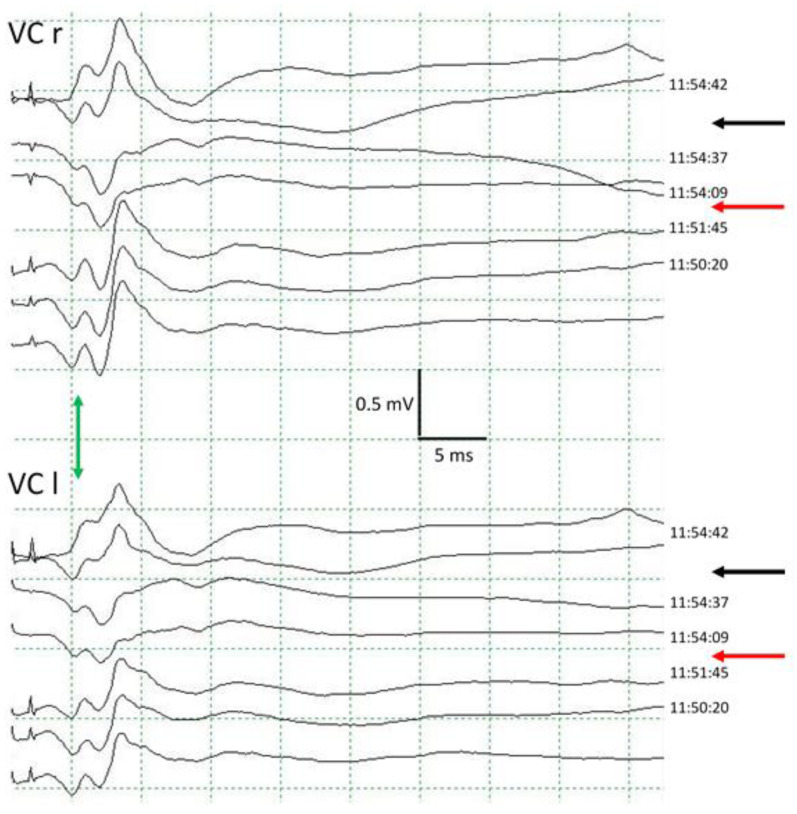
PAMC reduction in vocal cords due to traction of RLN (red arrows). Fast recovery after reducing the traction (black arrows). Note the persistence of the early PAMC corresponding to the superior laryngeal nerve (green arrows). VC: vocal cord. r: right. l: left.

RLN injury represents the most important complication in thyroid and parathyroid surgery. Its incidence is about 10% [7]. The identification of the RLN has always been the main objective for its preservation during surgery, but either due to its distortion or anatomical variants, its location is not always possible to determine. Its macroscopic anatomical integrity also does not always guarantee normal nerve function, and it is, therefore, necessary to wait for the immediate postoperative period to confirm normal VC function; a circumstance not without the potential for unpleasant surprises. The introduction of IOM in this type of intervention has not been exempt from initial difficulties due to the possible limitations on benefits, as well as unnecessary increases in surgical time [8,9,10,11]. However, results published in recent years have demonstrated the efficacy of IOM, its reliability, and the help it provides to surgeons in identifying and solving intraoperative problems [5,12,13,14,15,16,17,18,19,20,21]. The use of intraoperative monitoring of the recurrent laryngeal nerve helps to reduce the incidence of phonatory disorders, mainly in cases of cancer where lymphadenectomy is also performed [22]. IOM provides information in real-time, increasing surgical safety in complex situations that can occur unexpectedly. IOM helps in the identification of the recurrent and superior laryngeal nerve during dissection, thereby differentiating nervous and non-nervous tissue. IOM helps to prevent the development of definitive RLN lesions, and has prognostic value. Of equal importance, its use provides legal support in cases of postoperative sequelae [2,23,24,25]. An important fact that must be kept in mind when assessing the usefulness of IOM is that it is capable of detecting and preventing injuries that can develop progressively over time due to mechanisms such as traction (diffuse type 2 injury), as they can then be reversed at the end of the intervention through the use of modified surgical maneuvers. However, injuries that appear abruptly, due to transection, clamping, ligation, or thermal mechanisms (type 1 focal lesion) can only be detected using IOM, and not prevented; although, IOM does allow for reversing maneuvers to be used (for example, the removing clip) to avoid further damage to the nerve [5] (Figure 7). There are many international scientific societies that recommend the use of IOM, but there are no medical or surgical guidelines that advise against its use [2].

For the monitoring of the RLN, apart from its visual identification, other techniques have been used both for nerve stimulation and response recording, and the most common of which being electrical stimulation in the surgical field. Electrical stimulation uses the generation of an acoustic signal, associated, or otherwise, with the visual recording of a wave generated in VC, and alerts the surgeon to the presence of nerve tissue. This technique allows the nerve to be identified, but it does not give any information as to the nerve’s functional normalcy and could even give false positives if an inadequate stimulation intensity is used and is thereby diffused through non-neural tissues [1]. This technique in isolation helps, but it is insufficient considering the contribution of other monitoring methods currently available. The use of vagal stimulators placed directly on the nerve is the most effective means of monitoring the integrity of the RLN, since it allows continuous monitoring of its function throughout the entire intervention [5,26,27,28]. However, this technique also has significant drawbacks, and it requires dissection of the carotid sheath for its placement on the vagus nerve, which must be conducted bilaterally in total thyroidectomies, requiring a larger skin incision, greater risk of iatrogenesis, increased surgical time, and possible complications [23,29,30,31]. Other techniques have been described for IOM in thyroid and parathyroid surgery, such as the recording of CMAP in the posterior cricoarytenoid muscle [32], using anterior laryngeal electrodes [32] or the study of the laryngeal adductor reflex through electrical stimulation of the laryngeal mucosa and recording of the reflex response of the VC using surface electrodes in the endotracheal tube [33,34,35,36]. Although this technique monitors the sensory and motor pathways of the laryngeal nerves, it does not allow direct response (CMAP) to be obtained in the VC, which are always more reliable.

The ESVNCT techniques that we present have been shown to be an effective alternative method to the use of direct vagal stimulation. Our series of 150 monitored RLNs has shown high reliability, with a sensitivity of 0.87 and specificity of 0.94, and a very high NPV (0.99) with only one false negative. Our study identified sixteen RLN alterations due to reduction or disappearance of the CMAP, seven of which were associated with postoperative positive symptoms (with severe total or partial reduction of the CMAP) and in the remaining nine, the patients did not present voice alterations (each displayed partial reduction of CMAP with intraoperative recovery). In this case, the PPV is low (0.44) since the early detection of nerve involvement allows the surgery to be interrupted and the surgical technique to be modified to promote nerve recovery and to prevent the possible injury from progressing or becoming permanent. In this regard, these vagal stimulation techniques have demonstrated their effectiveness in the prevention of neural injuries, and not only in their detection. There was only one false negative noted, involving the detection of a very severe lesion of the left RLN during a thyroidectomy, without significant variations of the right CMAP, resulting in laryngeal stridor with respiratory distress and a subsequent tracheostomy. One month later, however, right-VC function returned to normal. The technique is safe and has caused no side effects in any of our patients. The use of continuous vagus nerve stimulation is very limited in endoscopic and robotic thyroid surgery [37], and ESVNCT could be an effective and simpler alternative, without the need for new incisions or complex stimulation techniques or any other surgical interference. We have not found any discrepancies in the results between either vagus nerve stimulation techniques, and the results obtained are always superimposable.

A frequently raised point of controversy is the cost-effectiveness of IOM in thyroid surgery [37,38]. The cost of this stimulation technique is very low though, as two subdermal electrodes currently cost around 3€ and a monopolar EMG electrode adds roughly another 4€. This is indeed much less than that of the direct vagal stimulator which has a starting price of approximately 80€, allowing for the expenses in materials and supplies to be considerably reduced. The possible interference with the surgery is also minimal, since only 1–2 s are required for its performance, and on many occasions, the surgeon themself will request nerve stimulation before and immediately after performing a risky maneuver, thus making the IOM much more dynamic and participatory. All the surgeons who operated on the monitored patients have been satisfied with these stimulation techniques and with its lack of interference on their activity.

In relation to the morphology variations of the CMAP, this is a finding common to all IOM techniques that use a recording electrode in the tracheal tube. The movements caused by the surgical maneuvers can lead to displacement of the tube either through sliding or rotation, with modification of the CMAP. In these cases, it is important to perform a contralateral vagal stimulation, which will also show a change in the morphology of the CMAP, confirming that these variations are due to recording problems and not due to nerve damage.

**Figure 8:**
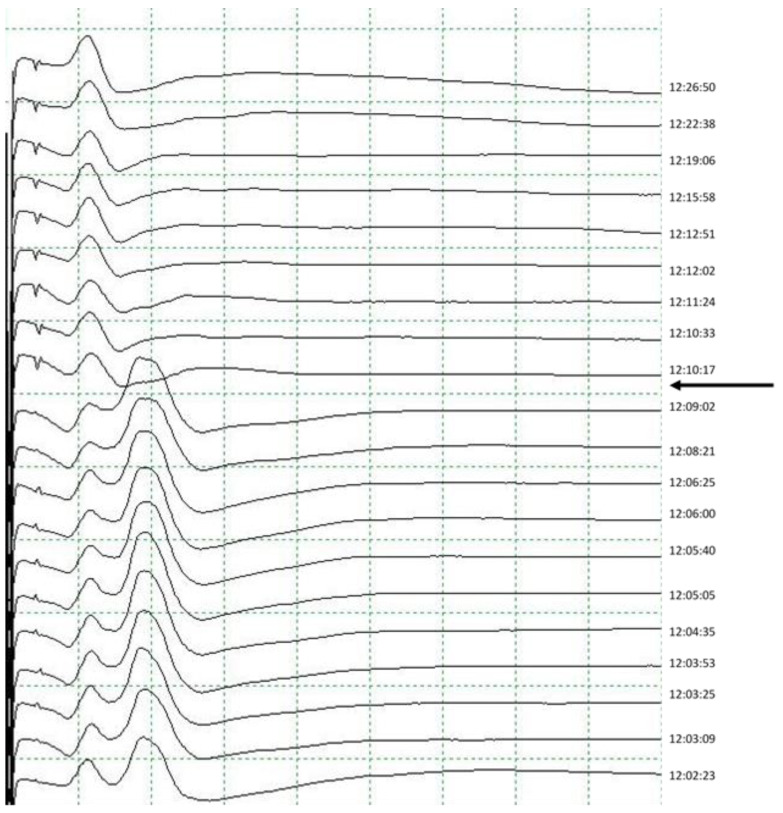
Complete lesion of the RLN with total disappearance of the PAMC (arrow) and without its recovery. The early PAMC corresponding to the superior laryngeal nerve remains unchanged.

One of the problems of IOM is establishing when a response should be considered pathological. Obviously, the most determining data is the complete disappearance of the CMAP (Figure 8), but amplitude drops of the CMAP are the main criterion used, having established that definitive amplitudes of less than 100 µV with stimulation of the vagus nerve or RLN in the surgical field should always be interpreted as pathological with little possibility of recovery and high probability of nerve injury [1,39,40], and it is very important that they have basal potentials greater than 500 µV [26]. Amplitude reductions greater than 50% and of unilateral appearance, and those not justifiable by displacement of the orotracheal tube should alert us to the possibility of nerve damage, requiring the notification of the surgeon and the modification of the maneuvers being performed. In all our patients with post-operative RLN injury, the CMAP disappeared or showed reductions greater than 54% at the end of surgery, while those in whom the maximum amplitude reduction was 47% at the end of the intervention had no sequelae. This indicates that a level of irreversible reduction greater than 50% would be critical to determine the existence of a lesion. This value coincides with those used previously [1,2,5] referring to CMAP amplitude reductions after direct vagal stimulation. Due to changes in the morphology of the CMAP, amplitude variations must not be correlated with the basal potential obtained at the beginning of surgery, but rather, with the last one obtained in the previous stimulation; underlining the importance of very frequent performance of nerve stimulation. Once the baseline CMAP is obtained, we continue monitoring with the stimulation that allows us to obtained a better response with the lowest stimulus intensity, reserving the other technique to confirm a possible loss of potential if it occurs. Usually, monopolar EMG electrode stimulation is the most effective. We have already said that with unilateral stimulation we always obtain a response in the theoretical recording of both VC, so we will use the best recording of the two to perform the monitoring.

The cEMG recording is a rarely used technique in thyroid IOM, generally because automatic stimulations are used that only assess CMAP latency and amplitude. However, the analysis of the EMG provides data of great value to prevent RLN injuries. One of the most common causes of injury is nerve traction during different moments of gland dissection [40]. When this happens, discharges are recorded in the EMG that alert us to the risk, the surgeon is then notified, traction ceases, and the EMG discharges disappear instantly. In this situation, we always perform vagal stimulation afterwards to check the functional status of the nerve. The combination of EMG and vagal stimulation is another method of continuous monitoring that helps us prevent the appearance of nerve injuries since the discharges in the EMG appear immediately with mechanical stimulation of the nerve, while the injury usually occurs when the traction is prolonged over time. Injuries produced acutely by heat, clipping, or nerve laceration are not usually accompanied by discharges in EMG.

The main limitation of the technique is failure of the recording electrode intraoperatively. The anatomy in patients with a short and thick neck makes surgery and also stimulation of the vagus nerve difficult; although, in these cases we have also been able to perform effective monitoring. Another limitation is the one already indicated above and that is that the circular distribution of recording points make it very difficult to differentiate to record the separate activity generated in the VC on each side, with them both in spontaneous cEMG and in CMAP after unilateral nerve stimulation. One of the limitations of our study, and which should be present in future studies, is that it would be helpful to assess the potential benefits of nerve monitoring post-thyroidectomy aero digestive and voice disorders, in the order of the studies of Melfa et al. [41].

## 5. Conclusions

The number of thyroid surgeries has continued to increase in recent years. The IOM has proven effective in thyroid and parathyroid surgery to identify and prevent RLN injuries. The techniques commonly used are the mapping of the RLN and the vagus nerve in the surgical field in order to achieve their identification and postoperative functional control, as well as the continuous stimulation of the vagus nerve through periodic automatic stimulation, which allows for continuous monitoring of nerve function throughout the entire surgery. This last technique, despite its proven benefits, is not being widely used, possibly because it requires prior dissection of the vagus nerve, which would be bilateral in the case of complete thyroidectomies. The use of ESVNCT has, though, been proven to be a valid alternative, allowing for semi-continuous monitoring with similar results, but without the need to directly access the nerve itself. Its value is also increased when it is associated with VC EMG, that can detect discharges in real-time caused by stimulation of the RLN due to traction, the main mechanism of nerve injury.

## Data Availability

The data used to support the findings of the present study are available from the corresponding author upon request.
